# The Prognostic Significance of Metastatic Nodal Size in Non-surgical Patients With Esophageal Squamous Cell Carcinoma

**DOI:** 10.3389/fonc.2020.00523

**Published:** 2020-04-16

**Authors:** Zongxing Zhao, Yanan Zhang, Xin Wang, Peiliang Wang, Xiaotao Geng, Liqiong Zhu, Minghuan Li

**Affiliations:** ^1^School of Medicine, Shandong University, Jinan, China; ^2^Department of Radiation Oncology, Shandong Cancer Hospital and Institute, Jinan, China; ^3^Department of Radiation Oncology, Liaocheng People's Hospital, Liaocheng, China; ^4^Department of Health Care, Liaocheng People's Hospital, Liaocheng, China; ^5^Shandong First Medical University, Shandong Academy of Medical Sciences, Jinan, China

**Keywords:** prognosis, node size, esophageal squamous cell cancer, non-surgical, radiotherapy

## Abstract

**Background:** The present study aimed to determine the prognostic value of the size of metastatic lymph node (LN) in non-surgical patients with esophageal squamous cell carcinoma (ESCC).

**Methods:** Three hundred seventy-six ESCC patients treated with definitive (chemo-) radiotherapy from January 2013 to March 2016 were reviewed. We analyzed potential associations of metastatic nodal size with responses, patterns of failure, and survival. Log-rank testing and Cox proportional hazards regression models were used to assess the impact of the clinical factors on survival.

**Results:** The 3-years over survival (OS) rates following a median follow-up of 28.2 months were 53.2, 46.2, 35.5, and 22.7% for the N0 group, the >0.5 to ≤1 cm group, the >1 to ≤2 cm group, and the >2 cm group, respectively. The progression-free survival (PFS) rates for 2 years were 50.9, 44.2, 26.6, and 23.4% for the N0 group, the >0.5 to ≤1 cm group, the >1 to ≤2 cm group, and the >2 cm group, respectively. The objective response rates (ORR) for the 280 patients with metastatic LNs were 43.1% for the LN >0.5 to ≤1 cm group, 46.9% for the LN >1 to ≤2 cm group, and 25.5% for the LN ≥2 cm group. The LN >2 cm group had the worst ORR of the three groups with LNs. Gross tumor volume (GTV) failure was the most common failure pattern, followed by distant failure and out of GTV LN failure, with incidences of 47.9% (180 of 376), 42% (158 of 376), and 13.8% (52 of 376), respectively. Nodal size correlated statistically with GTV failure and distant failure but not with out-of-GTV nodal failure. After adjusting for age, sex, T category, Primary tumor location, and CRT, the size of metastatic LNs was an independent prognostic factor for OS and PFS in multivariate analyses.

**Conclusions:** Nodal size is one of prognostic factors for non-surgical patients with ESCC and correlated statistically with GTV failure and distant failure.

## Introduction

Esophageal cancer (EC) was ranked seventh in terms of incidence and sixth in terms of mortality globally in 2018 (1). In parts of Asia, Squamous cell carcinoma (SCC) usually comprises over 90% of all esophageal carcinomas. Esophageal squamous cell carcinoma (ESCC) patients who are unfit for surgery or some medications are often treated with a combined-modality treatment with radiotherapy plus concurrent chemotherapy (2, 3). For this reason, a conformal intensity-modulated radiation therapy (IMRT) was recently established to facilitate dose modifications to reducing treatment-related toxicities (4).

Many surgical studies have suggested previously that lymph node metastasis influences the clinical course of EC (5–8). Currently the 8th American Joint Committee on Cancer (AJCC) tumor-node-metastasis staging system is applied to define the N categories in reference to the number of metastatic lymph nodes. However, this system is generally controversial for patients with non-surgical EC (8). It is not good enough to consider only the number of metastatic lymph nodes for radiation oncologists to develop treatment decision-making and evaluate prognosis. Published data has reported that Gross tumor volume (GTV) is the major prognostic factor for non-surgical patients with ESCC (9). Bulky lymph node metastasis is a particular pattern that was included in the N classification for head and neck carcinoma, and non-surgical therapy has adopted it as an important modality. This pattern is relevant in other kinds of malignancies, but its role in esophageal cancer patients is still unclear. Here, we investigated how the size of the metastatic lymph node (LN) affect the prognosis of thoracic ESCC to guide appropriate design of treatments.

## Materials and Methods

### Patients

We reviewed the cases of patients with biopsy-proven ESCC without distant metastasis. A total of 376 patients, who received definitive (chemo-) radiotherapy at Shandong Cancer Hospital from January 2013 to March 2016, were included in the study. Clinical variables, such as age, staging, tumor location, gender, survival outcomes, and failure patterns, were recorded. The patients with Tis-T1 stage received radical radiotherapy and were excluded. This study was approved by the medical ethics committees of Shandong Cancer Hospital.

### Criteria for LN Metastasis

During clinical assessment, all patients were screened by esophageal ultrasound (EUS), enhanced computed tomography (CT), barium swallow, and other underwent pretreatment staging workups, like fluorodeoxyglucose-positron emission tomography (FDG-PET) scan. We defined positive LN as a short-axis length >1 cm on CT or by a short-axis diameter of the pericardial angle, paraesophageal, tracheoesophageal sulcus, abdominal LN >5 mm (10). We defined LNs as positive if one of the following criteria: if the maximum standard uptake value (SUVmax) exceeded the background blood pool activity estimated in the normal thoracic aorta using FDG-PET or round shape, size ≥1 cm, clearly visible borders or hypoechoic pattern using EUS. We calculated the nodal size along the largest node based on images. Patients were classified into four groups based on the size of metastatic LNs (N0, >0.5 to ≤1 cm, >1 to ≤2 cm, >2 cm) according to lymph node imaging. The bulky LN was defined as the size of metastatic LN >2 cm.

### Treatment

All patients were treated with IMRT when the patient's health condition was allowed. GTV was assessed on the basis of pretreatment staging workups. The clinical target volume (CTV) was considered as the radial margins of 0.8 cm and the caudal as well as cranial margins of 3 cm of a primary tumor and regional lymph node area. A total dose of 50.4–66 Gy fractioned for 28–33 times was delivered using 6MV-X rays which could cover 95% PTV. Two hundred thirty-six patients were given concurrent platinum-based chemotherapy.

### Result Assessment and Follow-Up

The Response Evaluation Criteria in Solid Tumors (RECIST 1.0) system was applied to assess the LNs response in terms of complete response (CR), progressive disease (PD) partial response (PR), and stable disease (SD), for target lesions, CR, SD, and PD for non-target lesions (11). By definition, CR was considered as no FDG-avid lesions or disappearance of involved lymph node in our study. The objective response rates (ORR) was regarded as the sum of PR rate and CR rate. Patients were followed up with physical examination, including endoscopy, CT scans, and barium swallow, beginning from at 1 month at the end of therapy, followed by every 3 months in the first 2 years after treatment, and every 6 months thereafter until loss of follow-up or death. We characterized patterns of failure according to sites of failure and GTV failure (original LN and primary tumor), out of GTV LN failure and distant failure. Progression-free survival (PFS) and Overall survival (OS) were considered as the duration from the initial diagnosis.

### Statistical Analysis

All data analyses were performed using SPSS 23.0 (SPSS, Chicago, IL, USA). The Kaplan–Meier method was used to estimate survival rate. Characteristics of all participants were compared using Chi-square tests. Log-rank testing and Cox proportional hazards regression models were applied to determine associations between overall survival and clinical factors and between progression-free survival and clinical factors. A *p* < 0.05 was regarded as statistically significant.

## Results

### Clinical Characteristics of the Participants

Table 1 shows the patient characteristics. Overall, the 376 patients were followed up for a median duration of 28.2 months (range, 2–74 months), The median age of subjects was 62 years (range, 43–83 years). Among all the patients, 280 had metastatic LNs, with the median size of LNs = 1.1 cm (range, 0.5–7.1cm). The >0.5 to ≤1 cm group contained the most patients, followed chronologically by the N0 group, the >1 to ≤2 cm group, and the >2 cm group, with incidences of 36.4% (137 of 376), 25.5% (96 of 376), 25.5% (96 of 376), and 12.5% (47 of 376), respectively.

**Table 1:** Patient characteristics and univariate analysis of prognostic factors.

**Variables**	**Number of patients (%)**	**Median survival (*m*)**	**3-years survival rate (%)**	***P***
Age (years)				0.337
<60	108 (28.7%)	32.0	46.5%	
≥60	268 (71.3%)	27.8	40.5%	
Sex				0.186
Male	296 (78.7%)	28.0	40.1%	
Female	80 (21.3%)	32.6	50.3%	
Primary tumor location				<0.001
Upper	179 (47.6%)	41.5	55.7%	
Middle	136 (36.2%)	26.0	35.1%	
Lower	61 (16.2%)	16.0	21.3%	
T category				<0.001
T2	78 (20.7%)	49.0	55.0%	
T3	230 (61.2%)	28.0	42.4%	
T4	68 (18.1%)	17.5	22.8%	
No. of LNs				<0.001
0	96 (25.5%)	46.0	53.2%	
1–2	160 (44.4%)	36.0	49.1%	
3–6	103 (27.4%)	18.0	26.8%	
≥7	17 (4.5%)	13.0	0	
Size of LNs				<0.001
0	96 (25.5%)	46.0	53.2%	
>0.5 to ≤1 cm	137 (36.4%)	32.0	46.2%	
>1 to ≤2 cm	96 (25.5%)	24.8	35.5%	
>2 cm	47 (12.5%)	17.0	22.7%	
Treatment				0.013
CRT	236 (62.8%)	33.0	47.0%	
RT alone	140 (37.2%)	25.6	34.2%	

*LNs, lymph nodes; CRT, chemoradiotherapy; RT, chemoradiotherapy*.

### Response and Patterns of Failure

Based on LNs' responses, patients with metastatic LNs were divided into 2 separate groups: the PR+CR group and SD+PD group. The ORR of LNs for the 280 patients with metastatic LN were 43.1% for the LNs >0.5 to ≤1 cm group, 46.9% for the LNs >1 to ≤2 cm group, and 25.5% for the LNs ≥2 cm group. As shown in Table 2, the LNs ≥2 cm group had the worst ORR among the three groups (>0.5 to ≤1 cm group vs. ≥2 cm group: *P* = 0.038, LNs >1 to ≤2 cm group vs. ≥2 cm group: *P* = 0.018). Also, there were no significant differences between the >0.5 to ≤1 cm group and the LNs >1 to ≤2 cm group (*P* = 0.594).

**Table 2:** Correlation between size and responses of LN.

**Groups**	**PR+CR**	**SD+PD**	**Compared groups**	***P*-value**
>0.5 to ≤1 cm	59 (43.1%)	78 (56.9%)	>1 to ≤2 cm	0.594
			>2 cm	0.038
>1 to ≤2 cm	45 (46.9%)	51 (53.1%)	>2 cm	0.018
>2 cm	12 (25.5%)	35 (74.5%)		

**Figure 1:**
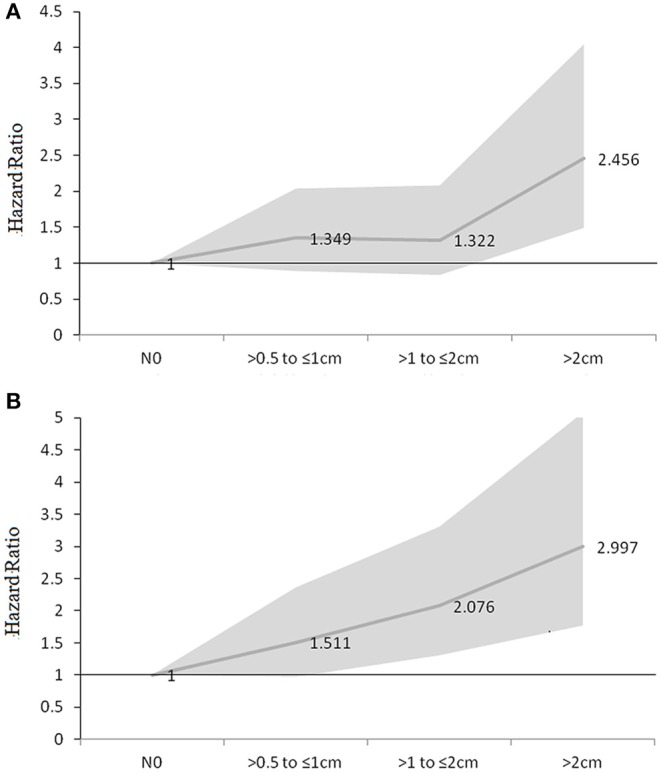
HR of GTV failure **(A)** and distant failure **(B)** for different size of LNs (HR adjusted for age, gender, tumor location, and T-category by 8th AJCC system).

Our analyses revealed that GTV failure was the most common failure pattern, followed by distant failure and out of GTV LN failure, with incidences of 47.9% (180 of 376), 42% (158 of 376), and 13.8% (52 of 376), respectively. Seventy-six patients (20%) had no evidence of disease. The number of failures in different sizes of LN in groups is illustrated in Table 3. Nodal size correlated statistically with local failure and distant failure but not with new regional LN failure. After adjusting age, gender, tumor location, and T-category, there was no significant difference in hazards ratio (HR) for GTV failure between the LN >0.5 to ≤1 cm group (HR, 1.313; 95%CI, 0.881–1.957), the LNs >1 to ≤2 cm group (HR, 1.462; 95%CI, 0.952–2.245) and the N0 group (HR, 1), but patients with LN >2 cm (HR, 2.198; 95%CI, 1.360–3.552) had a significant increase in HR in multivariate analyses. Significant differences were recorded in HR for distant failure between the LNs >1 to ≤2 cm group (HR, 2.076; 95%CI, 1.304–3.306), the LN >2 cm group (HR, 2.997; 95%CI, 1.768–5.081), and the N0 group (HR, 1). However, the hazards ratios (HR) for distant failure for patients in the >0.5 to ≤1 cm group (HR, 1.511; 95%CI, 0.967–2.36) was similar to that of patients with N0 (HR, 1) (Figure 1).

**Table 3:** Correlation between size and patterns of failure.

**Patterns of failure**	**Size**				***P*-Value**
	**NO**	**>0.5 to ≤1 cm**	**>1 to ≤2 cm**	**>2 cm**	
GTV failure					0.041
YES	35	61	40	30	
NO	61	76	58	17	
Distant failure					0.024
YES	31	55	45	27	
NO	65	82	51	20	
Out of GTV LN failure					0.759
YES	20	23	21	10	
NO	76	114	75	37	

### Survival

**Figure 2:**
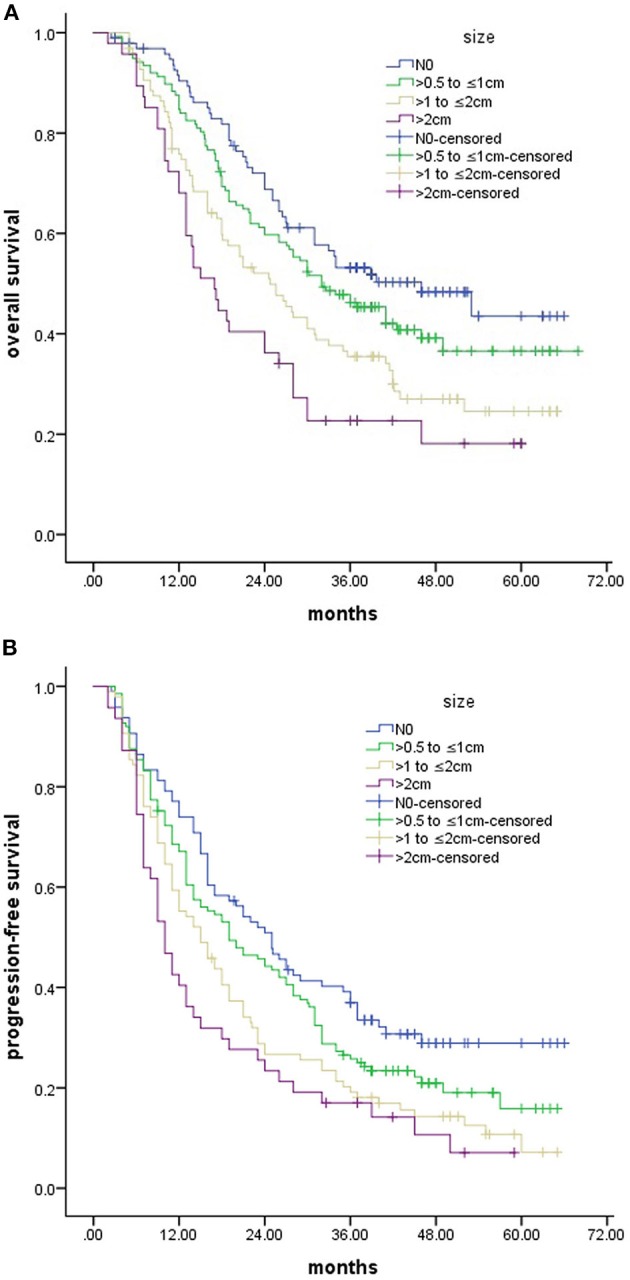
OS **(A)** and PFS **(B)** for patients stratified according to sizes of LNs.

The median survival time for all patients was 29.5 months, with a 1-year OS rate of 81.7%, 3-years OS rate of 42.3%, and 5-years OS rate of 33%, respectively. Kaplan–Meier analyses revealed significant differences in the OS (*P* < 0.001) and PFS (*P* < 0.001) based on the sizes of the LNs involved (Figure 2). The 3-years OS rates for were 53.2, 46.2, 35.5, and 22.7% for the N0 group, the >0.5 to ≤1 cm group, the >1 to ≤2 cm group, and the >2 cm group, with corresponding median survival times of 46.0, 32.0, 24.8, and 17.0 months, respectively. The median PFS time was 16.8 months (95% CI: 14.4–19.5 months). The 2-years PFS rates were 50.9, 44.2, 26.6, and 23.4% for the N0 group, the >0.5 to ≤1 cm group, the >1 to ≤2 cm group, and the >2 cm group.

**Figure 3:**
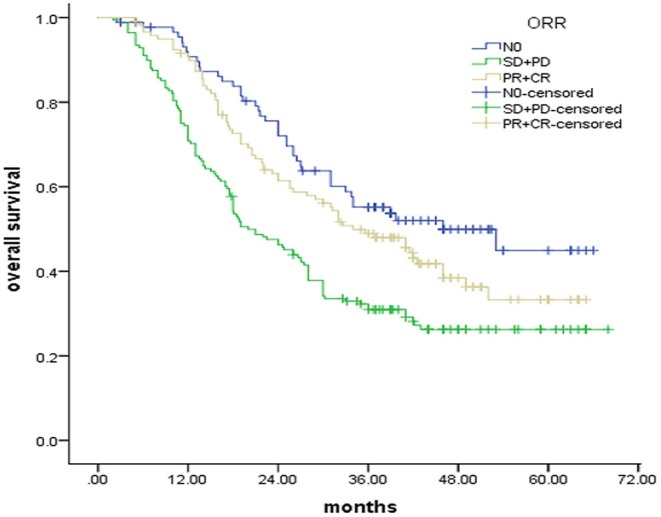
OS for patients stratified according to response of LNs.

To explore the influence of the responses of LNs on survival, we performed a Kaplan–Meier analysis to compare the PR + CR group and the SD+PD group. Significant differences were found between the two groups (*P* = 0.003, Figure 3). The responses of LNs had a positive prognostic significance on the 3-years survival period (48.9% for the PR + CR group vs. 31.0% for the SD + PD group) and median survival times (34.0 months for the PR + CR group vs. 20.0 months for the SD + PD group). Interestingly, the Kaplan–Meier analysis revealed that the PR+CR group had a comparable survival to that of the N0 group (*p* = 0.128, Figure 3).

### Univariate and Multivariate Analyses of Prognostic Factors

The T stage, tumor location, N category, CRT, and size of metastatic LNs were strongly linked to prognosis in the univariate survival analysis (Table 1). The multivariate analyses for survival were presented in Table 4. After adjusting for age, sex, *T* category, Primary tumor location, and CRT, the size of metastatic LNs was an independent prognostic factor for any of the two endpoints.

**Table 4:** Summary of multivariate cox regression analysis for the prognosis of patients with ESCC.

**End point**	**Prognostic factor**	**Hazard ratio**	**95% CI**	***P***
**OS**				
	Age(<60 vs. ≥60)	0.931	0.673–1.288	0.667
	Sex(Male vs. Female)	0.825	0.587–1.160	0.268
	Primary tumor location(baseline, Upper)			0.001
	Middle	1.422	1.056–1.914	0.020
	Lower	1.927	1.342–2.768	0.000
	T category (baseline, T2)			0.006
	T3	1.316	0.903–1.919	0.153
	T4	1.990	1.283–3.086	0.002
	Size of LNs (baseline, N0)			0.002
	>0.5 to ≤1 cm	1.309	0.906–1.893	0.152
	>1 to ≤2 cm	1.838	1.236–2.733	0.003
	>2 cm	2.116	1.345–3.329	0.001
	Treatment (CRT vs. RT alone)	0.669	0.510–0.878	0.004
**PFS**				
	Age (<60 vs. ≥60)	0.829	0.628–1.094	0.185
	Sex (Male vs. Female)	0.931	0.696–1.246	0.630
	Primary tumor location(baseline, upper)			0.001
	Middle	1.381	1.069–1.786	0.014
	Lower	1.804	1.301–2.500	0.000
	T category (baseline, T2)			0.044
	T3	1.276	0.965–1.711	0.061
	T4	1.528	1.040–2.246	0.031
	Size of LNs (baseline, N0)			0.019
	>0.5 to ≤1 cm	1.267	0.926–1.734	0.139
	>1 to ≤2 cm	1.581	1.126–2.219	0.008
	>2 cm	1.740	1.159–2.614	0.006
	Treatment (CRT vs. RT alone)	0.754	0.621–0.936	0.010

*LNs, lymph nodes; CI, confidence interval; CRT, chemoradiotherapy; RT, radiotherapy*.

## Discussion

According to the 8th edition AJCC system, N-classification for patients with EC is performed on the basis of the number of metastatic LNs. However, it is challenging to determine the exact number of metastasis LNs, especially in patients with non-surgical. Chen et al. found that the GTV of primary esophageal cancer and metastatic LNs may serve as a prognostic factor (9). They retrospectively analyzed 178 EC patients treated with radiotherapy and indicated that patients whose GTV>39.41 cm^3^ had significantly worse survival when compared to those whose GTV ≤ 39.41 cm^3^. So, we think that more variables should be taken into account as prognostic implications for radiation oncologists to develop individual radiotherapy strategies for ESCC patients treated with definitive radiotherapy. The nodal size was included as one of the variables used for N staging in the head and neck cancer. Previous studies have suggested that the short axis of the pre-therapeutic node in patients with ESCC undergoing neoadjuvant treatment followed by esophagectomy is a prognostic factor (12, 13). In our study, we assessed potential associations between the size of metastatic LNs and survival in non-surgical patients with ESCC. We found that PFS and OS rates differed between patients with different sizes of LNs using univariate analyses. Bulky LNs was associated with poorer prognosis in ESCC patients undergoing definitive chemo-radiotherapy.

For patients with EC who receive neoadjuvant chemoradiation, pathologic complete response (pCR) is associated with lower rates of PFS and better OS (14, 15). In our study, patients with clinical PR and CR of involved nodes had a similar survival rate with N0 patients (*p* = 0.128). Our data suggest that response to CRT was associated with significantly improved survival in non-surgical patients with esophageal carcinoma. Some studies have suggested that metabolic tumor volume (MTV), one of the FDG-PET parameters, predicts the response of metastatic LNs for patients with ESCC who receive dCRT (16, 17). Zhu et al. divided 143 metastatic LNs from 59 patients treated with dCRT into 4 groups and found that MTV-G4 (which had the largest MTV) had the lowest CR rate (18). In our study, we also found that bulky LNs had a lower ORR than the other groups, which may be one reason why the bulky LN had the worst OS. Another reason may have been that the metastatic nodal size was interrelated significantly with the extranodal neoplastic spread (ENS). One research reported finding ENS in 27.2% of patients with the primary head and neck SCC with nodes measuring <2 cm, in 55.8% of those measuring 2 to 4 cm, and in 100% of those measuring >5 cm (19). ENS can be employed in the diagnosis of malignant nodes and also a prognostic factor that influences treatment (20).

The optimal radiotherapy dose for patients with localized ESCC undergoing CRT remains controversial. According to the INT 0123 phase III trial, 50.40 Gy is the recommended CRT dose in the North America guideline (3). Some studies have suggested that dose escalation may improve local control and OS for thoracic ESCC with a high locoregional control rate (21–24). Zhang et al. found that patients with stage II or III EC patients administered with a total dose >51 Gy showed markedly better DFS and OS (24). In Asian, SCC is the main histological type and 60 Gy is frequently used in clinical practice. However, most of these studies have focused on the dose of a primary tumor and not for metastatic LNs. There is little evidence for the effects of irradiation-dose escalation IMRT for LNs in patients with EC. Zhu et al. found that an escalated dose (59.7 Gy) could improve the CR rate of metastatic LNs with higher MTV (18). In our study, patients with bulky LNs had lower ORR and worst survival. Higher dose radiotherapy may lead to better local control or survival for patients with bulky LNs, pending further in-depth studies.

In our study, GTV failure was the most common failure pattern, followed by distant failure and out of GTV LN failure. We found that the HR for distant failure and GTV failure for bulky LN metastasis was higher than those for other groups. Similar results were observed by Mine S et al. who reviewed 222 patients who had undergone neoadjuvant treatment followed by esophagectomy for ESCC and found that size of metastatic LNs was significantly associated with distant failure but not LN failure (13). For ESCC patients CRT, GTV failure and distant failure were the most predominant patterns which lowered the survival. According to Welsh et al.'s review of 239 patients who underwent definitive chemoradiation therapy using elective nodal irradiation(ENI), most local failures were found in the GTV (25). In another study, Zhang et al. reviewed 76 patients treated with involved-field irradiation (IFI) and found that 40 patients (53.75%) had experienced GTV failure, 31 (41.25%) had distant metastasis, and 23 (30%) had initially uninvolved LN failure. Patients with initially uninvolved LN failure had similar survival when compared with those without initially uninvolved LN failure (26). Therefore, for ENI or IFI, GTV failure was the main pattern of local failure in patients with local advanced ESCC.

Chemoradiotherapy is the current standard treatment for non-surgical patients with EC. However, there is no global consensus on the definition of the LN CTV. Considering that micrometastases should be controlled, ENI had been implemented in several phase III trials (Radiation Therapy Oncology Group [RTOG] 85–01, 94–05) (2, 3). The primary tumor recurrence and distant failure are the most common failure patterns for EC patients with definitive CRT. Majority of the studies on ESCC have focused on the comparison between ENI and IFI in EC. Li et al. performed a retrospective evaluation of the failure patterns of 56 subjects with T4M0 and found only 1 patient had experienced isolated elective nodal failure (27). In our study, patients with bulky LN had lower response rates and worst survival. And bulky LN correlated statistically with GTV failure and distant failure but not with an out-of-GTV regional nodal failure. When we develop local treatment strategies for patients with bulky LNs, the higher radiation dose should be given and IFI should be performed to minimize treatment toxicity.

Our study has some drawbacks. First, it is a retrospective study carried out at a single institution. The potential biases and the relatively small patient numbers in the T1 and T4 subgroups may limit statistical power. Second, the influence of histologic differentiation on prognosis was not analyzed because it would have been difficult to obtain in most patients confirmed by biopsy. Third, lymph nodes metastases were diagnosed according to non-invasive pretreatment staging workups, including EUS, CT, and FDG-PET, rather than histologic diagnosis. However, a strength of this study is that we investigated the prognostic value of the size of metastatic LN for cases with ESCC treated with definitive (chemo-)radiotherapy, which was rare in previous research. In contrast with the previous studies of surgical cases, we also evaluated the association of the size of metastatic LN with clinical response and failure patterns to provide more information for the treatment strategy. Moreover, distant failure was also common in our study, especially in the bulky LN metastasis subgroup. Thus, efforts to improve OS may have to await improvements in systemic therapies for the successful control of distant diseases.

## Conclusions

The sizes of LNs, especially bulky nodes, were the independent prognostic factors for non-surgical patients with ESCC and should be considered as prognostic factors for radiation oncologists. Patients with bulky LNs may need a higher radiation dose, and alteration of involved target volume may be feasible. Further observations from large-scale studies are needed to verify these results.

## Data Availability Statement

The data used to support the findings of this study are available from the corresponding author upon request.

## Ethics Statement

The studies involving human participants were reviewed and approved by Shandong Cancer Hospital Affiliated to Shandong University. The patients/participants provided their written informed consent to participate in this study. Written informed consent was obtained from the individual(s) for the publication of any potentially identifiable images or data included in this article.

## Author Contributions

ZZ and ML analyzed the data and drafted the manuscript. YZ, PW, XW, XG, and LZ participated in data collection.

### Conflict of Interest

The authors declare that the research was conducted in the absence of any commercial or financial relationships that could be construed as a potential conflict of interest.

## Abbreviations

ESCC: esophageal squamous cell carcinoma
EC: esophageal carcinoma
CRT: chemoradiotherapy
LNs: lymph nodes
FDG-PET: fluorodeoxyglucose-positron emission tomography
AJCC: American Joint Committee on Cancer
CT: computed tomography
CTV: clinical target volume
EUS: endoscopic ultrasound
GTV: gross tumor volume
PTV: planning target volume
RT: radiotherapy
HR: hazards ratio
PD: progressive disease
SD: stable disease
ORR: objective response rates
PR: partial response
CR: complete response
OS: overall survival
PFS: progression-free survival.
